# Contrasting phylogeographic patterns and demographic history in closely related species of *Daphnia longispina* group (Crustacea: Cladocera) with focus on North-Eastern Eurasia

**DOI:** 10.1371/journal.pone.0207347

**Published:** 2018-11-14

**Authors:** Elena I. Zuykova, Evgeniy P. Simonov, Nickolai A. Bochkarev, Sergey A. Abramov, Natalia G. Sheveleva, Alexey A. Kotov

**Affiliations:** 1 Laboratory for Ecology of Vertebrate Communities, Institute of Systematics and Ecology of Animals of Siberian Branch of the Russian Academy of Sciences, Novosibirsk, Russia; 2 Laboratory of Biology of Aquatic Invertebrates, Limnological Institute of Siberian Branch of the Russian Academy of Sciences, Irkutsk, Russia; 3 Laboratory of Aquatic Ecology and Invasions, A.N. Severtsov Institute of Ecology and Evolution of Russian Academy of Sciences, Moscow, Russia; National Cheng Kung University, TAIWAN

## Abstract

Species with large geographic distributions present a challenge for phylogeographic studies due to the logistic difficulties of obtaining adequate samples. *Daphnia* O.F. Müller (Anomopoda: Daphniidae) is a model genus for evolutionary biology and ecology, but many regions such as the remote areas of Siberia, remain poorly studied. Here we examined genetic polymorphism in the ribosomal *12S* and the protein-coding *ND2* mitochondrial genes of three closely related taxa of the *Daphnia* (*Daphnia*) *longispina* complex, namely *D*. *galeata* Sars, *D*. *longispina* O.F. Müller and *D*. *dentifera* Forbes. We estimated the phylogenetic relationships among these taxa based on a concatenated alignment of these two genes. Using sequences from the present study and those available in GenBank, we investigated the geographic distributions of the mitochondrial haplotypes of these species and proposed an evolutionary scenario for each taxon. Network structures, haplotype distribution patterns, and *F*_ST_ values indicated significant differences in the evolutionary history of the examined species. Our analysis of *D*. *galeata* populations confirmed its recent and fast expansion, without a previous phase of a strong population disconnection. In contrast, the high haplotype diversity in *D*. *dentifera* and *D*. *longispina* could be explained by the survival of different phylogroups in several glacial refugia located in different geographic regions. For all studied species, maximum haplotype diversity was recorded in the remote regions of Siberia–lakes of the Yenisei River and Transbaikalia. Our study is an important step in our understanding of the evolutionary history of the *Daphnia longispina* group and provides further evidence of the biogeographic significance of Siberia for freshwater taxa.

## Introduction

Water fleas (Crustacea: Cladocera) are intensively studied model organisms for molecular and evolutionary biologists. The most significant progress in cladoceran taxonomy was achieved at the end of the 20th–beginning of the 21st century, when several families of the orders Anomopoda Sars and Ctenopoda Sars were globally revised based on morphological characters [1–5], while many other groups are waiting for taxonomic revision.

During the last decades, molecular methods have been intensively used in studies of cladoceran diversity [6–12]. It was shown that many "widely distributed taxa" of cladocerans are in fact grоups of more locally distributed species [11, 13–18]. Molecular methods were successfully tested for resolving some problems of the "traditional" morphology-based taxonomy [19–21]. It is obvious to date that a combination of morphological and molecular methods is the best way for further development of taxonomy, such approach is tested for different genera of Cladocera [14, 22–25]. In general, investigations based on molecular methods have significantly improved our knowledge of cladoceran biodiversity.

*Daphnia* O.F. Müller (Anomopoda: Daphniidae) is the most species rich genus among the cladocerans [26, 27] and is used as a general model for evolutionary biology [28, 29]. At the same time, it is a well-known example of a taxon with very confused taxonomy [26]. Especially confused is the *D*. *longispina* species group, containing many widely distributed taxa dominating in the plankton of large and small lakes [11, 30]. The morphology of many daphniids is still described insufficiently, if not completely inadequately [27]. Recently the taxonomic status of some species of the *longispina*-group was elucidated by application of molecular methods, which allowed a reconstruction of phylogenetic relationships between different taxa and populations and even describe evolutionary processes within them [11, 31–34]. Hybridization between several mitochondrial lineages also was demonstrated [35–39] making such efforts much more complicated. A mito-nuclear discordance is also known in this group [40].

A very important source of information on the evolutionary history of the daphniids, as well as other freshwater organisms, is phylogeography [41], a genetic variant of historical biogeography [42]. This approach has been applied to several daphniid groups since the pioneering publications in late 1990’s [13]. Different evolutionary scenarios were revealed for different daphniid taxa, and several speciation models were proposed for cladocerans. It was shown that a rapid recent expansion is characteristic of both *Daphnia pulex* and *D*. *galeata*, resulting in a situation in which shared haplotypes of these taxa are present in distant regions [38, 43, 44]. A large number of the haplotypes in *D*. *dentifera* and *D*. *longispina* confirmed that most antique lineages of these taxa survived in some refugia during the periods of maximum glaciation [38, 44, 45]. Phylogeographic investigations of *Daphnia* were mainly concentrated on well-accessed regions: North America [13, 46] and Europe [10, 16, 47]. Only during the last decade, were Eastern Palearctic regions as Japan, the Far East of Russia [38, 48, 49] and China [34, 44, 50] included in such studies.

Most recent studies of the phylogeography and evolutionary history of *Daphnia* (as well as other cladocerans) have a sampling bias–a huge region–Siberia–is not studied in the majority of these works. For example, such taxa as *D*. *umbra* and *D*. *dentifera* were surprisingly detected in some remote lakes of Eastern Siberia, and other mitochondrial lineages are also revealed there [51–53]. Such findings of earlier undetected species allow us to hypothesize that divergent lineages of the *D*. *longispina* complex in Siberia could potentially represent separate biological species, and their evolutionary history could be different from that in others regions, e.g. Europe, North America and Japan. Such differences could be related to differences in geological and climatic events during the last glacial maximum and previous Pleistocene glacial cycles [48, 54, 55, 56]. However, most populations of *Daphnia* from remote regions of Siberia remain untouched by molecular geneticists, although it was shown for *D*. *umbra* that the Siberian populations exhibited clear mitochondrial divergence from the Arctic populations [21]. Accordingly, previous global phylogeographic conclusions on *Daphnia* species are vulnerable to criticism. Only recently have global trans-Palearctic phylogeographic studies with adequate coverage of Siberia been conducted for other cladoceran groups: *Chydorus* [14, 18], *Moina* and *Daphnia magna* [17, 57].

The aim of this study is to investigate the geographic distribution of the mitochondrial haplotypes of three closely related species of the *Daphnia* (*Daphnia*) *longispina* complex inhabiting different water bodies of Siberia: *D*. *galeata* Sars, *D*. *longispina* O.F. Müller and *D*. *dentifera* Forbes. We propose an evolutionary scenario for each taxon and analyze differences between these three taxa. To do this, we studied the genetic polymorphism of ribosomal *12S* and protein-coding *ND2* mitochondrial genes and reconstructed phylogenetic relationships between these taxa based on concatenated sequences of the two genes.

## Materials and methods

### Ethics statement

The study did not involve any endangered or protected species. Field collection in Russia was carried out by our team or by colleagues as part of a governmental project "Ecology and biodiversity of aquatic ecosystems and invasions of alien species" (No 0109-2014-0008 for 2015–2017) and the Federal Fundamental Scientific Research Program for 2013–2020 No VI.51.1.9. (AAAA-A16-116121410119-4), with governmental permission to collect samples from public property. Sampling in the natural reserves of Russia (Azas Federal Natural Reserve; Baikalo-Lenskiy Reserve and Pribaikalskiy National Park) was conducted with special permissions of their Administration, which is also thanked for assistance during the sampling. A part of samples was taken at the Field Stations of the Institute of Systematics and Ecology of Animals, and we thank Dr A.K. Yurlov, the Director of Chany Field Station, and Dr I.I. Chupin, the Director of Teletskoe Lake Field Station, for their help. Mongolian samples were collected by the Joint Russian-Mongolian Complex Biological Expedition with permission of the Ministry of Nature, Environment and Tourism of Mongolia.

### Sampling

**Fig 1:**
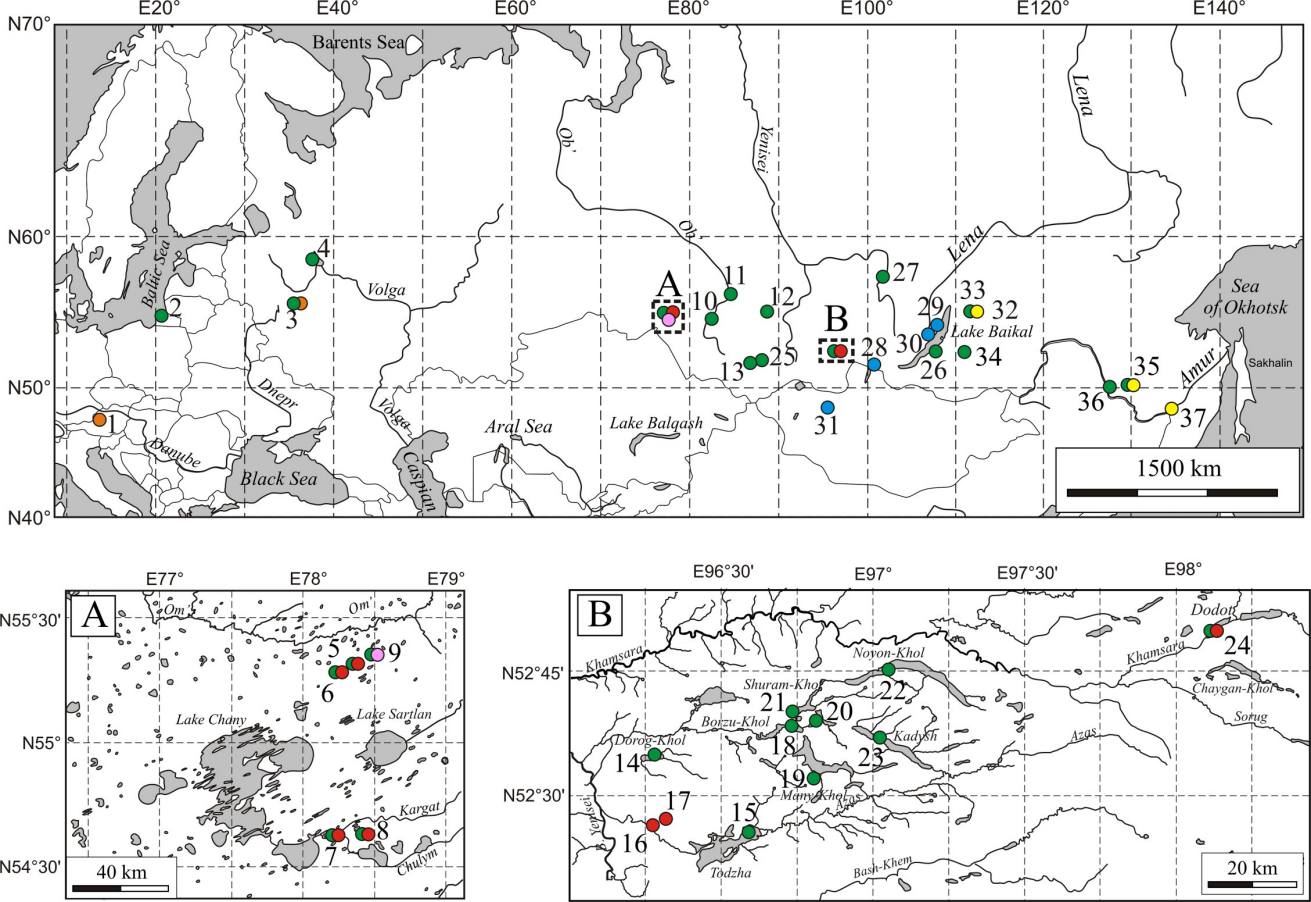
Map of the *Daphnia* sampling sites. Upper panel–a total map. Lower panel–A, the Chany Lake basin; B, the Todzha Depression. Circles of different colors correspond to different species, namely: green, *D*. *galeata*; red, *D*. *longispina*; orange, *D*. cf. *hyalina*; blue, *D*. *dentifera*; black, *D*. cf. *longispina*; yellow, *D*. *cristata*. The base maps are freely available from public domain https://www.d-maps.com, and from https://www.openstreetmap.org under the Open Database License v.1 and under the Creative Commons Attribution-ShareAlike 4.0.

Original samples were collected by the Juday-type (125 μm mesh size) and Apstein-type (250 μm mesh size) plankton nets during the summer seasons of 2004−2013 from 35 water bodies in the European part of Russia (Glubokoe Lake, Rybinsk Reservoir, the Couronian Lagoon), the southern part of Siberia (the Chany and Baikal lake basins, Novosibirsk Reservoir, Teletskoye Lake, lakes of the Todzha Depression and Transbaikalia), the Russian Far East and also in a single lake in Austria (Hallstättersee) and a single lake in Mongolia (Zhaakhan) (Fig 1, S1 Table).

Immediately after collecting, the samples were fixed in 96% ethanol and then stored in the freezer at –20°C. Prior to DNA extraction each specimen was photographed in lateral view using an AxioScan microscope (Carl Zeiss, Germany, under 10× and 50×) for documentation of its body and head shape. Each specimen was identified to species level according to existing keys [58–60]. Identification of *D*. *galeata* based on morphological characters in most cases was not difficult, while the status of some specimens provisionally placed to *D*. *longispina* s. str., *D*. *hyalina* and *D*. *dentifera* caused doubts, and finally such specimens were identified based on genetic data only.

*D*. *hyalina* is a taxon with unresolved taxonomic status, it is considered either as a lacustrine morphotype of *D*. *longispina* [11], or a separate species [30, 60]. In this study, we mixed all *D*. *hyalina*-like sequences with the *D*. *longispina* s. str. sequences, because the sequence number of the former was too small for any adequate conclusions on *D*. *hyalina*’s status.

Representative samples are maintained in the collections of E.I. Zuykova, at the Institute of Systematics and Ecology of Animals of Siberian Branch of the Russian Academy of Sciences, Novosibirsk, Russia.

### DNA extraction and sequencing

Total DNA was extracted from single ethanol-preserved *Daphnia* individuals with a 5% suspension of Chelex 100 resin (Bio-Rad, USA). The 20 μl PCR reaction consisted of 2–5 μl of genomic DNA (10–20 ng per 100 μl of the DNA homogenate), 0.5 μM dNTPs, 2 μl 10× PCR buffer (10 mM Tris–HCl, pH 8.3, 50 mM KCl), 2.5 mM MgCl_2_, 0.5 μM of each primer, 1 unit of *Thermus aquaticus* DNA polymerase (*Taq*-pol) and double-distilled H_2_O. The *12S* rRNA (*12S*) gene was amplified according to the protocol described in Colbourne & Hebert [9], and *ND2* gene according to the protocol of Ishida et al. [49].

The PCR products were separated on 1% agarose (Low EEO Standart agarose, BIOZYM, Russia) in the presence of ethidium bromide and photographed under UV light. The amplified products were purified using a kit from BIOSILICA (Novosibirsk, Russia) and both strands were sequenced on an ABI 3130xl automated capillary sequencer (Applied Biosystems) with the ABI Prism BigDye Terminator Cycle Sequencing Ready Reaction Kit 3.1 at the SB RAS Genomics Core Facility (Novosibirsk, Russia, http://sequest.niboch.nsc.ru). The DNA sequences were automatically aligned using the ClustalW algorithm [61].

82 sequences of 508–511 bp for *D*. *galeata*, 528–529 bp for *D*. *longispina*/*D*. *hyalina* and 525–528 bp for *D*. *dentifera* of the *12S* gene and 99 sequences of 718 bp of the *ND2* gene were newly obtained and deposited into the GenBank database (for accession numbers see S1 Table). The earlier obtained sequences [51, 52, 62] and orthological sequences for the *D*. *galeata*, *D*. *longispina* and *D*. *dentifera* species from the GenBank database [6, 10, 11, 38, 46, 51, 52, 62] were also included into the genetic analyses. In total, 277 nucleotide sequences for the *12S* gene and 132 nucleotide sequences for the *ND2* gene were analyzed.

### Phylogenetic analyses

For reconstruction of the phylogenetic relationships between the species of the *D*. *longispina* complex only original *12S* and *ND2* sequences were used. The *12S* rDNA sequences were aligned using PRANKSTER software which is a graphical front-end to the multiple sequence alignment program PRANK [63, 64]. Sequences of *ND2* gene were aligned manually and checked for unexpected stop codons using BioEdit v.7.0 [65]. All gaps and poorly aligned positions were detected and removed in the *12S* alignment using the Gblocks program (online version 0.91b; [66]) with smaller final blocks allowed.

Unique haplotypes were selected from concatenated dataset (*ND2* and *12S*) for downstream phylogenetic analysis with maximum likelihood (ML) and Bayesian inference (BI). We determined the best fit models of nucleotide substitution and optimal partitioning scheme (by marker and by codon position for *ND2*) in concatenated dataset using PartitionFinder v.1.1.1 [67] under Bayesian Information Criterion (BIC). ML search was performed using RAxML v.8.2.4 [68] with GTR+G model and independent parameter estimation for the following three subsets within the partition (BIC = 12 383.43; lnL = -5 753.68): *ND2* codon position 2; *ND2* codon position 3; *ND2* codon position 1 + *12S* rDNA. The tree support was accessed with the rapid-bootstrapping algorithm using 1000 non-parametric bootstrap replicates. Bayesian analysis was performed with MrBayes v.3.2.1 [69] under the following partition (BIC = 12 361.02; lnL = -5 749.66): *12S* rDNA (HKY+G); *ND2* codon position 1 (K80+G); *ND2* codon position 2 (GTR+G); *ND2* codon position 3 (GTR). Two simultaneous runs with four Markov chains each were run for 1 × 10^6^ generations and sampled every 500 generations. The first 25% of generations were discarded as burn-in. Convergence of runs was assessed by examination of the average standard deviation of split frequencies and the potential scale reduction factor. In addition, stationarity was confirmed by examining posterior probability, log likelihood, and all model parameters by the effective sample sizes (ESSs) in the program Tracer v.1.6 [70]. The phylogenetic trees resulting in ML and BI analyses were visualised and edited using FigTree v.1.4 [71] and Dendroscope v.3.4.4 [72]. The sequences of *D*. *cristata* were used for outgroup rooting of the phylogenetic tree.

### Polymorphism and divergence of the mitochondrial DNA

The mitochondrial DNA polymorphism at the species level for *D*. *dentifera*, *D*. *galeata* and *D*. *longispina* was estimated separately for *12S* and *ND2* genes. The following parameters were calculated: number of polymorphic sites (segregating, *S*), number of haplotypes (*h*), haplotype diversity with standard deviation (*H*_d_), and nucleotide diversity with standard deviation (π). The calculations were performed with DnaSP v.5.10 [73]. Divergence and gene flow between the geographical *Daphnia* populations was estimated using the index *F*_ST_ based on the *12S* sequences in Arlequin v.3.5.1 [74]. In total from four to eight geographical *Daphnia* populations were allocated in this analysis.

### Haplotype distribution and demographic history

We detected signatures of demographic history for the Siberian *D*. *galeata* and *D*. *longispina* populations using several approaches, namely: indexes of polymorphism sequences, Tajima’s *D* and Fu’s *F*_S_ neutrality tests, the structure of median-joining networks and mismatch distributions.

The haplotype networks were constructed by the median-joining method (MJ) [75] using Network v.4.5 (available on www.fluxus-engineering.com) based on the *12S* sequences for the *D*. *dentifera*, *D*. *galeata* and *D*. *longispina* species, and based on the *ND2* sequences for *D*. *galeata* only.

The neutrality tests of Fu’s *F*_S_ [76] and Tajima’s *D* [77] also were calculated for species and geographical clades with DnaSP v.5.10. The significance of these tests was proved using the coalescent simulation with 10 000 permutations.

Additionally, to investigate the demographic history of the Siberian populations of *D*. *galeata* and *D*. *longispina*, the mismatch distribution (MMD) was used [78, 79] using Arlequin v.3.5.1. In general, multi-modal MMD testifies demographic equilibrium of studied population, and uni-modal suggests recent demographic expansion [80, 81], or range expansions with a high migration activity between neighbor local populations [78, 79].

The demographic parameter Tau (τ) was used to estimate a time since expansion (*t*) with the equation τ = 2*ut*, where *u* = M_T_μ, M_T_ the number of nucleotides under study, and μ the mutation rate per generation [80]. The bootstrap approach (1000 replications) was used to test the observed data with the simulated data under the models of pure demographic expansion and spatial expansion by comparing the sum of squared deviations (SSD) between the observed (SSD_obs_) and simulated (SSD_sim_) data. The Harpending’s raggedness index (*r*) was used to test for a deviation from unimodality of the mismatch distribution. The significance of the estimated parameters was also obtained from the corresponding *P* values. The 95%-confidence intervals around τ, M and Theta were calculated with the bootstrap approach (1000 replications). The divergence rate of arthropod’s mtDNA was assumed as 2% per million years [14, 15, 82, 83] and 3 generations per years (as it was estimated for the alpine *D*. *longispina* populations, [45]). To avoid common errors in the results of mismatch distribution we follow the algorithm reported by Schenekar & Weiss [84]. The mismatch distribution for *D*. *galeata* was calculated on the *12S* and *ND2* genes separately.

The protocol of this investigation is available at protocols.io (dx.doi.org/10.17504/protocols.io.s7pehmn).

## Results

### Phylogeny based on *12S*+*ND2* sequences

**Fig 2:**
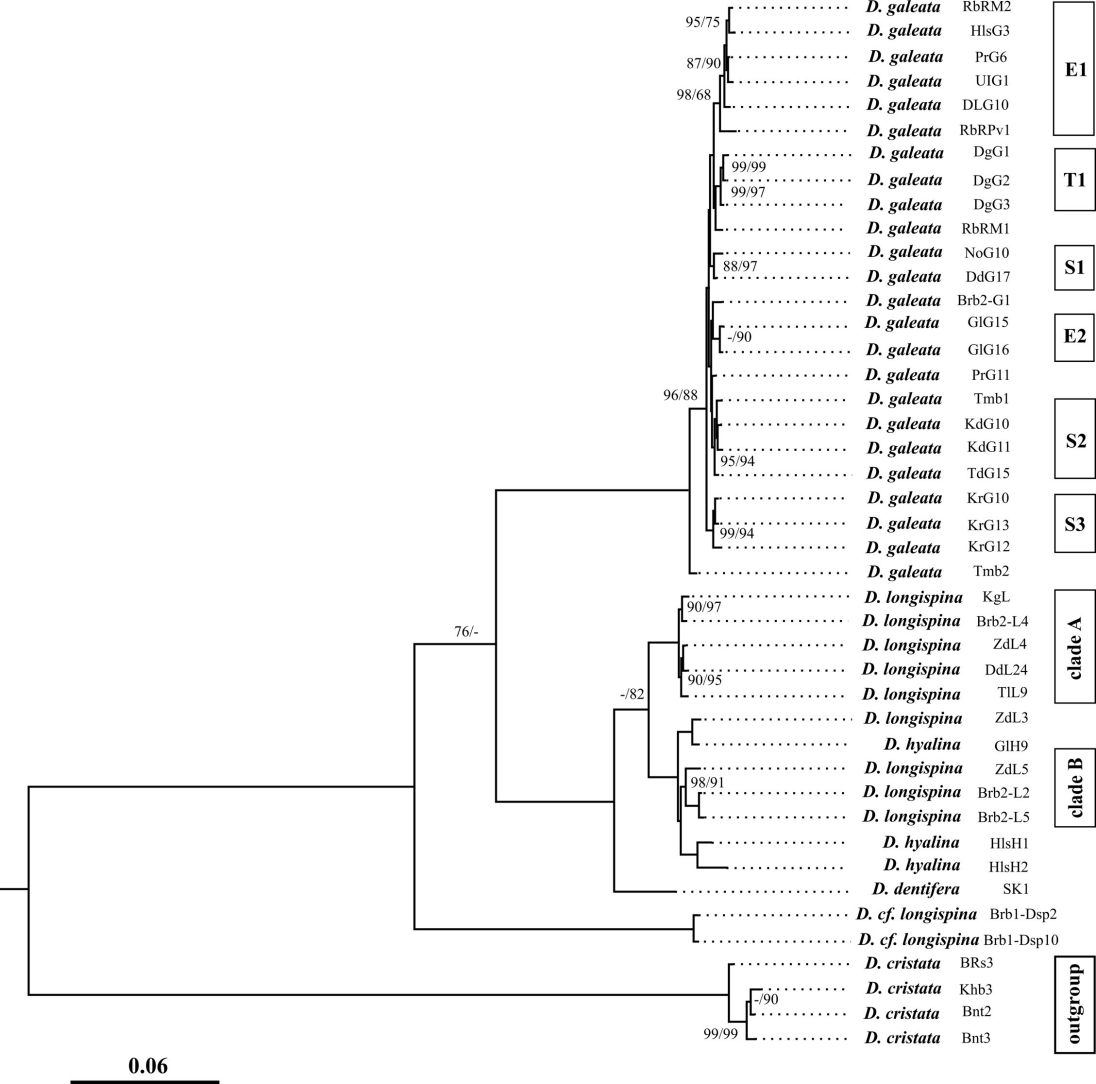
Bayesian phylogenetic consensus tree for *D*. *longispina* s.l. based on concatenated *ND2* and *12S* sequences (1319 bp). Bayesian posterior probabilities and bootstrap values from ML analysis expressed as a percentage (above 72%) are indicated. The scale is given in expected substitutions per site. E1 and E2, European *D*. *galeata* haplogroups; T1, Transbaikalian *D*. *galeata* haplogroup; S1-S3, Siberian *D*. *galeata* haplogroups; A and B clades correspond to different Siberian *D*. *longispina* haplogroups.

Our phylogenetic reconstruction based on the *12S*+*ND2* sequences reveals four major clades well-corresponded to the following species: *D*. *galeata*, *D*. *longispina*, *D*. *dentifera*, *D*. cf. *longispina* (plus the fifth clade is corresponded to *D*. *cristata*, an outgroup in our analysis) (Fig 2). All major clades are well-supported statistically.

Within *D*. *galeata* and *D*. *longispina* some divergent haplotypes form also well-recognized local geographical haplogroups with a high support (88–99%). Several such clades of *D*. *galeata* are revealed in Siberia, namely: the Kadysh, Dodot and Noyon-Khol lakes of the Todzha Depression in the Bolshoy Yenisei River basin (clades S1 and S2), Karakul Lake in the Bolshoy Abakan River basin (clade S3), Dorong Lake in Transbaikalia (T1). A single *D*. *galeata* haplotype from a pond near Blagoveshchensk in the Far East of Russia (Tmb1) contains in the clade S2. A single clade is mixed and consists of haplotypes from different geographical areas (clade E1, Fig 2).

All sequences of *D*. *longispina* are separated into two large divergent mitochondrial clades (although the latter have no significant statistic support) (Fig 2): (1) an exclusively Siberian clade A, and (2) a clade B widely distributed through whole territory of the Palearctic. Siberian clade A is represented by populations of *D*. *longispina* from distant Siberian water bodies: the Chany Lake basin (ZdL, Brb2-L, KgL), Teletskoye Lake in the Ob River basin (TlL) and Dodot Lake in the Yenisei River basin (DdL). Clade B is formed by the *D*. *longispina* haplotypes from the water bodies of the Chany Lake basin. The *D*. cf. *hyalina* haplotypes from European populations (Russia, GlH and Austria, HlsH) are close related to the *D*. *longispina* haplotypes of clade B.

All Siberian sequences of *D*. *dentifera* from two lakes of the Baikal basin are represented by a single haplotype (SK). *D*. *longispina* is clustered with *D*. *dentifera*, and then this group forms a clade with *D*. *galeata*. An undescribed *longispina*-like taxon (marked here as *D*. cf. *longispina*) is revealed in a single temporary pond in the Chany Lake basin, it forms a separate cluster, although having no good branch support.

### Mitochondrial DNA polymorphism

The level of genetic polymorphism of two mitochondrial genes is comparable in all studied species (Table 1), although both *H*_d_ and π values are somewhat higher in *D*. *dentifera* and *D*. *longispina* than in *D*. *galeata*. In the latter case, a level of haplotype (*H*_d_) and nucleotide (π) diversity is higher for the fragment of the protein-coding gene *ND2* as compared to *12S*. The Siberian clade A of *D*. *longispina* demonstrates very low nucleotide diversity in both genes. This clade also has a small number of haplotypes and polymorphic sites as compared to the Siberian clade B.

**Table 1:** Polymorphism of the *12S* and *ND2* genes of the mtDNA of studied *Daphnia* species and two Siberian *D*. *longispina* clades. Abbreviations: *n* (pop), number of sequenced *Daphnia* individuals and populations; *S*, number of polymorphic sites; *h*, number of haplotypes; *H*_d_, haplotype diversity; **π,** nucleotide diversity; st.d., standard deviation.

Species/Clade	*n* (pop)		*S*		*h*		*H*_d_ ± st.d.		π ± st.d.	
	*12S*	*ND2*	*12S*	*ND2*	*12S*	*ND2*	*12S*	*ND2*	*12S*	*ND2*
*D*. *galeata*	154 (57)	86 (32)	48	94	46	49	0.833±0.029	0.975±0.008	0.0044±0.0004	0.0091±0.0006
*D*. *longispina*	85 (46)	28 (11)	59	255	45	20	0.970±0.008	0.968±0.019	0.0142±0.0007	0.0957±0.0181
Siberian clade A	23	8	7	5	7	3	0.771±0.059	0.750±0.096	0.0022±0.0004	0.0031±0.0009
Siberian clade B	11	8	18	178	7	8	0.873±0.089	1.000±0.063	0.0081±0.0026	0.0782±0.0329
*D*. *dentifera*	38 (21)	18 (11)	38	65	27	13	0.962±0.021	0.941±0.041	0.0092± 0.0174	0.0167± 0.0031

### Geographical distribution of haplotypes

#### D. galeata

**Fig 3:**
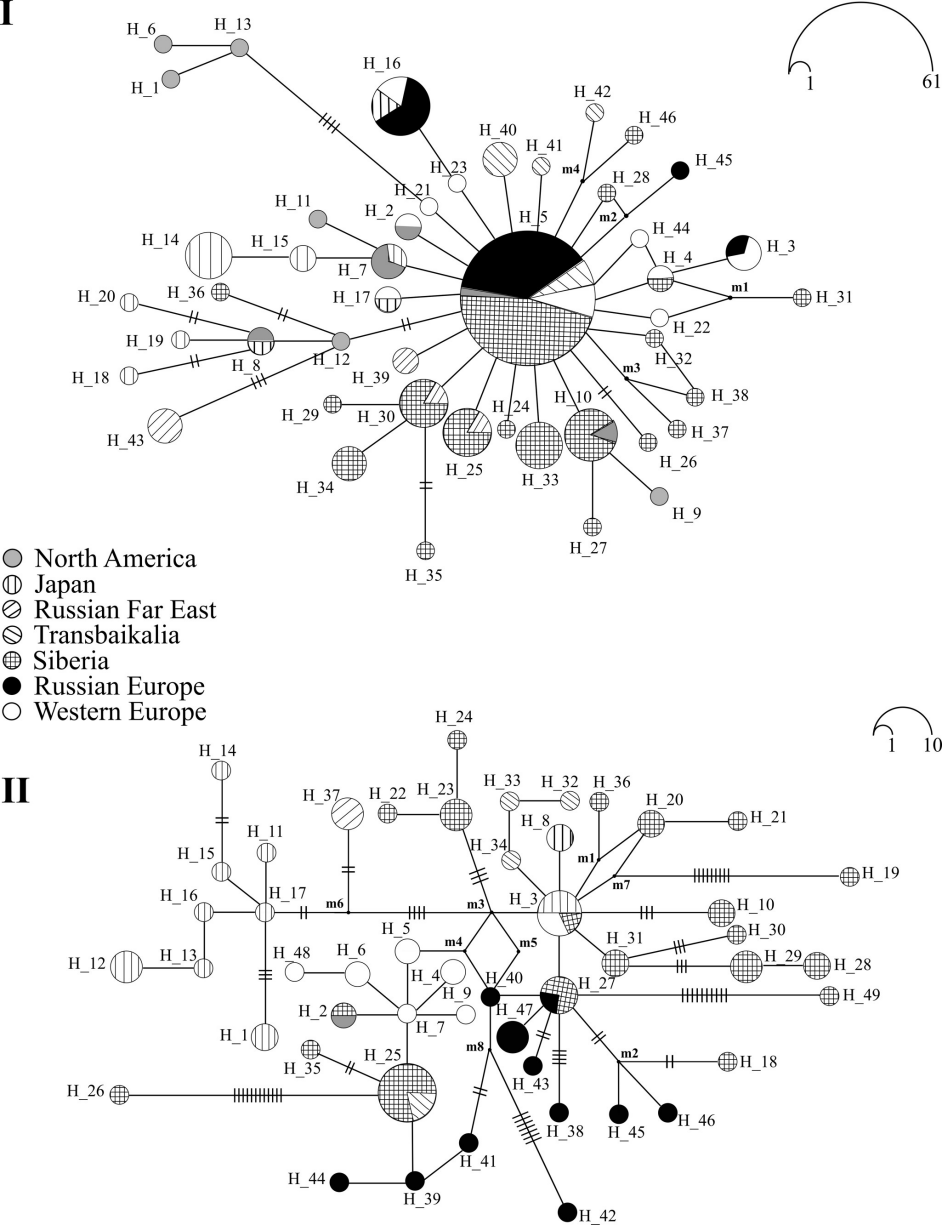
Median-joining haplotype *12S* (I) and *ND2* (II) networks for *Daphnia galeata*. The circle sizes correspond to relative haplotype frequencies (circular size scales are shown in the upper right corner); m1–m4 is median vectors. The numbers of mutations are labeled by lines for each branch (if not 1).

The *12S* based network of *D*. *galeata* haplotypes has a very characteristic star-like pattern. Its center is occupied by a single ancestral haplotype H5 (Fig 3I) which occurs in almost all regions of the Palearctic (including geographically distant regions): Western and Eastern Europe, Eastern Siberia, Transbaikalia, the Far East of Russia, Japan, China; it is also present in North America. Unique haplotypes of *D*. *galeata* are recorded in all studied regions; most divergent haplotypes are present in the water bodies of Japan, China and North America (H1, H6, H13, H18, H20, and H43), Siberia (H24, H27-H29, H31-H38, H46), some haplotypes are shared between Siberia and the Far East (H25, H30) and Siberia and Japan-China (H10).

Distribution pattern of *ND2* haplotypes in *D*. *galeata* is remarkably different from that for *12S* haplotypes, and network structure is much more reticulated and complicated (Fig 3II). Unique haplotypes are detected in each studied geographic region. Remarkably, no *ND2* haplotypes of *D*. *galeata* sharing between European Russia and Western Europe are found. Strongly divergent *ND2* haplotypes are detected in Siberia (H10, H19, H26, H28-H30, H49) and Eastern Europe (= European Russia) (H42). Maximum frequency is detected for the haplotype H25 from Siberia and Transbaikalia, this haplotype is closely related to those distributed in Japan, European Russia and Western Europe. Several star-like patterns are revealed within afore described network, their central haplotypes are detected in Siberia and Transbaikalia (H25), Siberia and Europe (H27), Siberia, Western Europe and North America (H3). Two central haplotypes are regional: H7 (Western Europe) and H17 (North America); several North American haplotypes (H1, H11-H17) are diverged from the latter forming a special regional cluster with a Far Eastern haplotype H37 as a sister clade.

#### D. longispina

**Fig 4:**
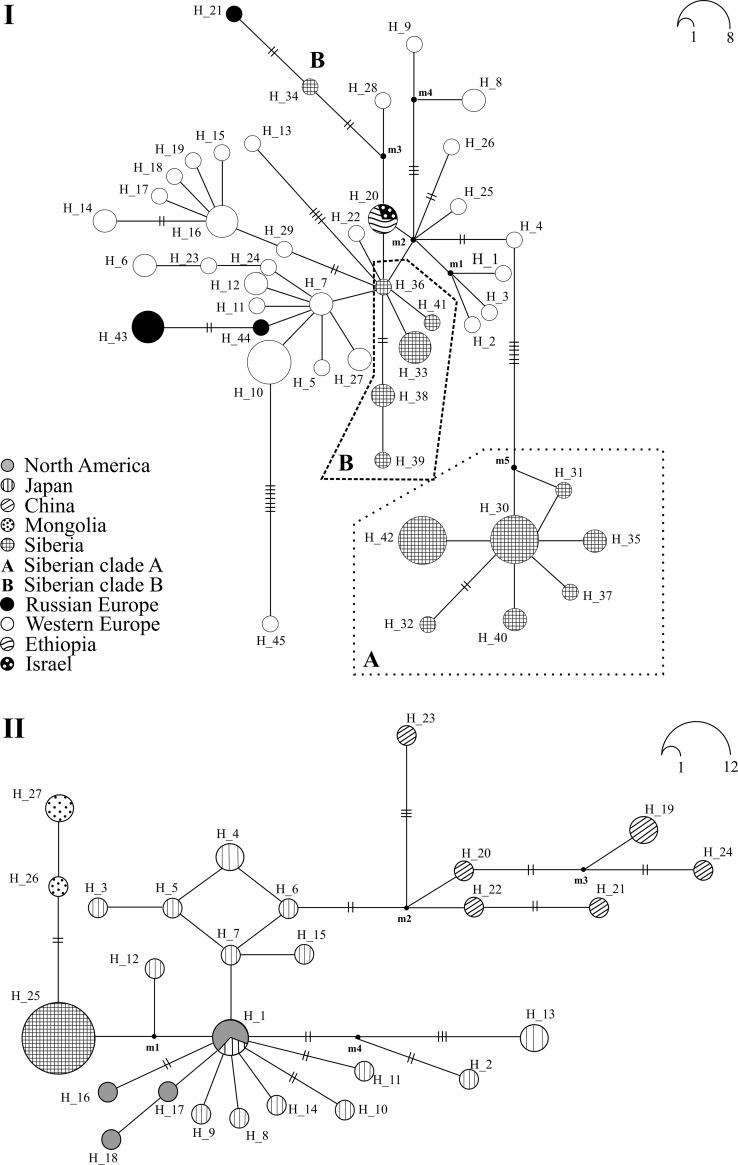
Median-joining *12S* haplotype networks for *Daphnia longispina* (I) and *Daphnia dentifera* (II). The legend corresponds to that in Fig 3.

Surprisingly, no single *12S* haplotype sharing for Siberian and European populations is found. Moreover, with a single exclusion of the haplotype H20 sharing between relatively distant regions (Israel and Ethiopia), all haplotypes of *D*. *longispina* are regional endemics (Fig 4I). Higher frequencies are registered for haplotypes H10 (Western Europe), H42 (Eastern Siberia) and H30 (Western and Eastern Siberia). Most divergent haplotypes are found in Western Europe (H8, H9, H45), Eastern Europe (H21, H34) and Western Siberia (H43). Several star-like shapes are revealed, with central haplotypes H7 (Western Europe), H16 (Western Europe), H30 (Siberia), H36 (Siberia) and a hypothesized haplotype m2.

Two well-recognized clades of Siberian populations, corresponding to the Siberian clade A and Siberian clade B from the *12S*+*ND2* tree are detected. The clade A with the central haplotype H30 is formed by populations from the Yenisei River basin and the Chany Lake basin (Eastern and Western Siberia). The haplotypes of the Siberian clade B are connected to the haplotypes from Western Europe through the central haplotype H36 (detected in Western Siberia only). Central haplotypes of two Siberian clades are interconnected each other through a Western European haplotype H4 and seven mutations. There is a third exclusively Siberian group, represented by a single haplotype H34, as it was noted above, it is strongly divergent and does not belong to any large clade.

#### D. dentifera

Two main clades of the *12S* haplotypes are revealed (Fig 4II). The first clade unites the haplotypes from North America and Eastern Eurasia (mainly Japan), and a Siberian-Mongolian cluster (H25-H27) is a part of the second clade. Central haplotype of a single well-recognized star-like shape (H1) occurs in Japan and North America, and the Siberian-Mongolian cluster is connected with the former through a hypothesized haplotype m1. The second, exclusively Chinese, clade unites most divergent haplotypes H19-H24.

### Demographic history

A major portion of the daphniid populations are characterized by negative values of Fu’s *F*_S_ and Tajima’s *D* for both studied mitochondrial genes (Table 2). Positive, although insignificant, values of Fu’s *F*_S_ for *ND2* and Tajima’s *D* for both *12S* and *ND2* are registered for a group uniting all Siberian population of *D*. *longispina*. In contrast to such "uniting" Siberian population, the Tajima’s *D* values are negative for *12S* in each Siberian clade (A and B) separately, and for *ND2* in the Siberian clade B.

**Table 2:** The neutrality tests for different *Daphnia* species.

Species/Population/Clades	Fu’s *F*_S_		Tajima’s *D*	
	*12S*	*ND2*	*12S*	*ND2*
***D*. *dentifera***	-19.321***	**-1.006**	**-1.666**	**-1.560**
North America	-0.848	-1.291	-0.562	-1.589
Japan	-10.608***	n.a.	-1.405	n.a.
China	-3.081*	–	-0.045	–
Baikal basin	n.a.	n.a.	n.a.	n.a.
***D*. *galeata***	-52.117***	-29.789***	-2.282**	-1.797*
North America	-0.296	-1.111	0.195	0.308
Japan	-5.358*	–	-0.393	–
Russian Far East	1.345	2.813	1.488	0.186
Transbaikalia	-2.376*	-0.304	-1.244	1.034
Siberia	-16.190***	-2.882	-2.218**	-1.299
Eastern (Russian) Europe	1.089	-3.851*	-0.51	-1.014
Western Europe	-5.263*	-2.055*	-0.993	-0.171
***D*. *longispina***	-24.432***	**3.936**	**-1.216**	**-0.254**
Siberia	-0.554	7.984*	0.067	1.086
Siberian clade A	-2.721	1.837	-1.237	0.756
Siberian clade B	-0.749	0.490	-1.384	-1.189
Eastern (Russian) Europe	1.020	n.a.	-1.367	n.a.
Western Europe	-17.841***	1.920	-1.720	-1.125

*P < 0.05

** *P* < 0.01

***P < 0.001

n.a., not available.

Pairwise *F*_ST_ values between the *D*. *galeata*, *D*. *longispina* and *D*. *dentifera* populations based on *12S* gene are represented in Tables 3–5. Highest values (> 0.5) of this index for *D*. *galeata* based on the *12S* gene are registered under pairwise comparison of Siberian, Transbaikalian and Eastern European populations with Chinese ones, and also comparing Eastern European populations with North American and Far Eastern ones (> 0.41) (Table 3). Highest values (> 0.7) of this index based on the *12S* gene for *D*. *longispina* are registered under pairwise comparison of the Siberian clade A with all other clades (Table 4); and Baikalian *D*. *dentifera* populations with all other populations (> 0.39, Table 5).

**Table 3:** Pairwise *F*_ST_ values between the populations of *D*. *galeata* based on *12S* gene of mtDNA.

Geographical population	North America	Japan	China	Russian Far East	Baikal-Transbaikalia	Siberia	Russian Europe	Western Europe
North America	–	0.02	0.10	0.01	0	0	0	0
Japan	0.14	–	0.21	0.06	0.01	0	0	0
China	0.33	0.03	–	0.11	0.01	0	0	0.01
Russian Far East	0.25	0.15	0.29	–	0	0	0	0
Baikal-Transbaikalia	0.31*	0.14	0.58	0.36*	–	0.03	0	0.01
Siberia	0.37*	0.19*	0.52	0.37*	0.03	–	0	0
Russian Europe	0.41*	0.26*	0.68*	0.48*	0.13	0.07*	–	0
Western Europe	0.27*	0.16*	0.43	0.32*	0.09	0.09*	0.02	–

* *P* ≤ 0.01

** *P* ≤ 0.001.

Standard error estimates are shown above the diagonal.

**Table 4:** Pairwise *F*_ST_ values between the populations and two Siberian clades of *D*. *longispina* based on the *12S* gene of mtDNA.

Geographical population/clade	Siberian clade A	Siberian clade B	Russian Europe
Siberian clade A	–	0	0
Siberian clade B	0.80**	–	0
Russian Europe	0.88**	0.38*	–
Western Europe	0.71**	0.09*	0.26*

* *P* ≤ 0.01

** *P* ≤ 0.001.

Standard error estimates are shown above the diagonal.

**Table 5:** Pairwise *F*_ST_ values between the populations of *D*. *dentifera* only based on the *12S* gene of mtDNA.

Geographical population	North America	Japan	China	Baikal basin
North America	–	0.16	0.18	0
Japan	0.03	–	0.08	0
China	0.07	0.03	–	0
Baikal basin	0.62*	0.39*	0.52*	–

* P ≤ 0.01

** *P* ≤ 0.001.

Standard error estimates are shown above the diagonal.

**Fig 5:**
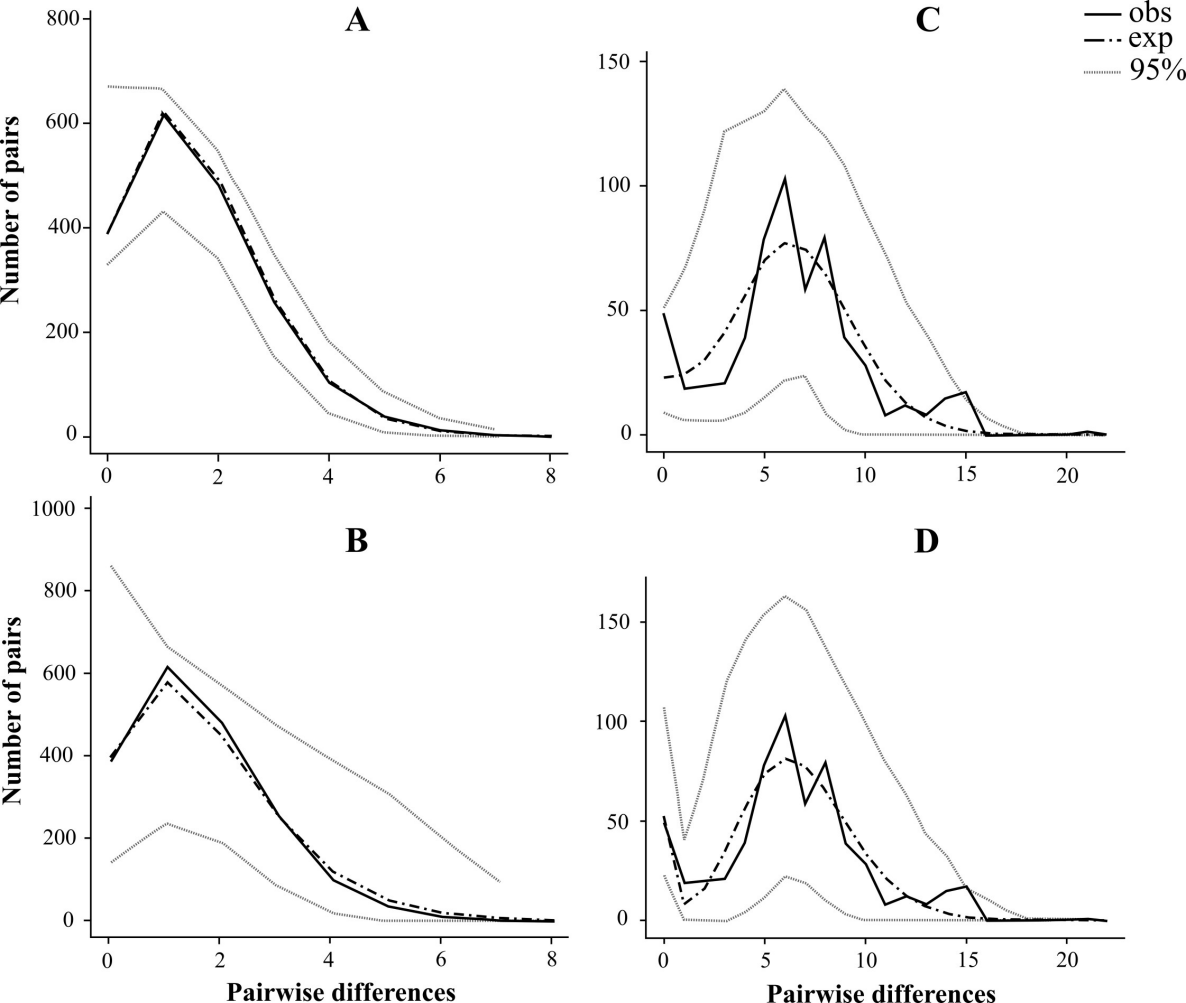
**Mismatch distribution of the Siberian populations of *Daphnia galeata* based on the *12S* (A and B) and *ND2* (C and D) sequences**. Black line represents the observed distribution. Dash and dot line and grey dotted lines represent expected distributions and the 95% credible interval for the sudden expansion model (A and C) and the spatial expansion model (B and D).

Mismatch distribution graphs are represented here only for the Siberian *D*. *galeata* and *D*. *longispina* populations. The MMD values computed for the Siberian *D*. *galeata* populations are different for two genes. Unimodal mismatch distribution for the *12S* gene is well-correlated with the sudden expansion model (SSD_obs_ = 0.000036, Table 6; Fig 5A and 5B). In contrast, bimodal mismatch distribution for *ND2* gene is better correlated with the spatial expansion model (SSD_obs_ = 0.00667, Fig 5C and 5D).

**Table 6:** Estimated parameters for expansion models of the Siberian *Daphnia galeata* and *Daphnia longispina* populations. SSD_obs_, sum of square deviation; *r*, Harpending’s raggedness index; τ, time of expansion; Theta, Theta 0, and Theta 1, mutation parameters; *M*, numbers of migrants; CI, 95% creditable interval.

Index	*D*. *galeata*		*D*. *longispina*		*D*. *longispina* (*12S* gene)
	*12S* gene	*ND2* gene	*12S* gene	Clade A	Clade B
**Sudden expansion model**					
SSD_obs_ (*P* value)	0.000026 (0.984)	0.00989 (0.287)	0.06489 (0.101)	0.01335 (0.210)	0.01935 (0.690)
*r* (*P* value)	0.04188 (0.059)	0.02363 (0.289)	0.05318 (0.405)	0.1247 (0.160)	0.02942 (0.928)
τ (95% CI)	1.590 (0.832–2.379)	7.086 (3.777–10.008)	13.387 (2.553–86.388)	1.328 (0.328–2.518)	3.992 (1.604–7.504)
Theta 0 (95% CI)	0 (0–0.875)	0 (0–2.257)	0 (0–4.205)	0 (0–0.032)	0.025 (0–2.791)
Theta 1 (95% CI)	313.153 (2.442–84.558)	25.0 (14.417–99999.0)	6.802 (2.719–35.620)	99999.0 (3.673–99999.0)	6.787 (3.328–99999.0)
**Spatial expansion model**					
SSD_obs_ (*P* value)	0.00077 (0.600)	0.00667 (0.781)	0.05036 (0.149)	0.01335 (0.170)	0.01687 (0.750)
*r* (*P* value)	0.04189 (0.185)	0.02363 (0.797)	0.05318 (0.731)	0.12476 (0.180)	0.02942 (0.930)
τ (95% CI)	1.073 (0.527–3.328)	6.010 (3.166–9.240)	10.710 (0.922–89.820)	1.327 (0.493–2.152)	1.787 (0.500–10.056)
Theta (95% CI)	0.622 (0.001–2.351)	0.745 (0.001–3.449)	3.524 (0.001–7.762)	0.001 (0.001–1.056)	2.406 (0.001–5.799)
*M*	3972.772 (2.587–4814.642)	9.917 (4.421–35.333)	0.916 (0.242–3498.822)	99999.0 (9.912–99999.0)	8.107 (0.669–99999.0)

**Fig 6:**
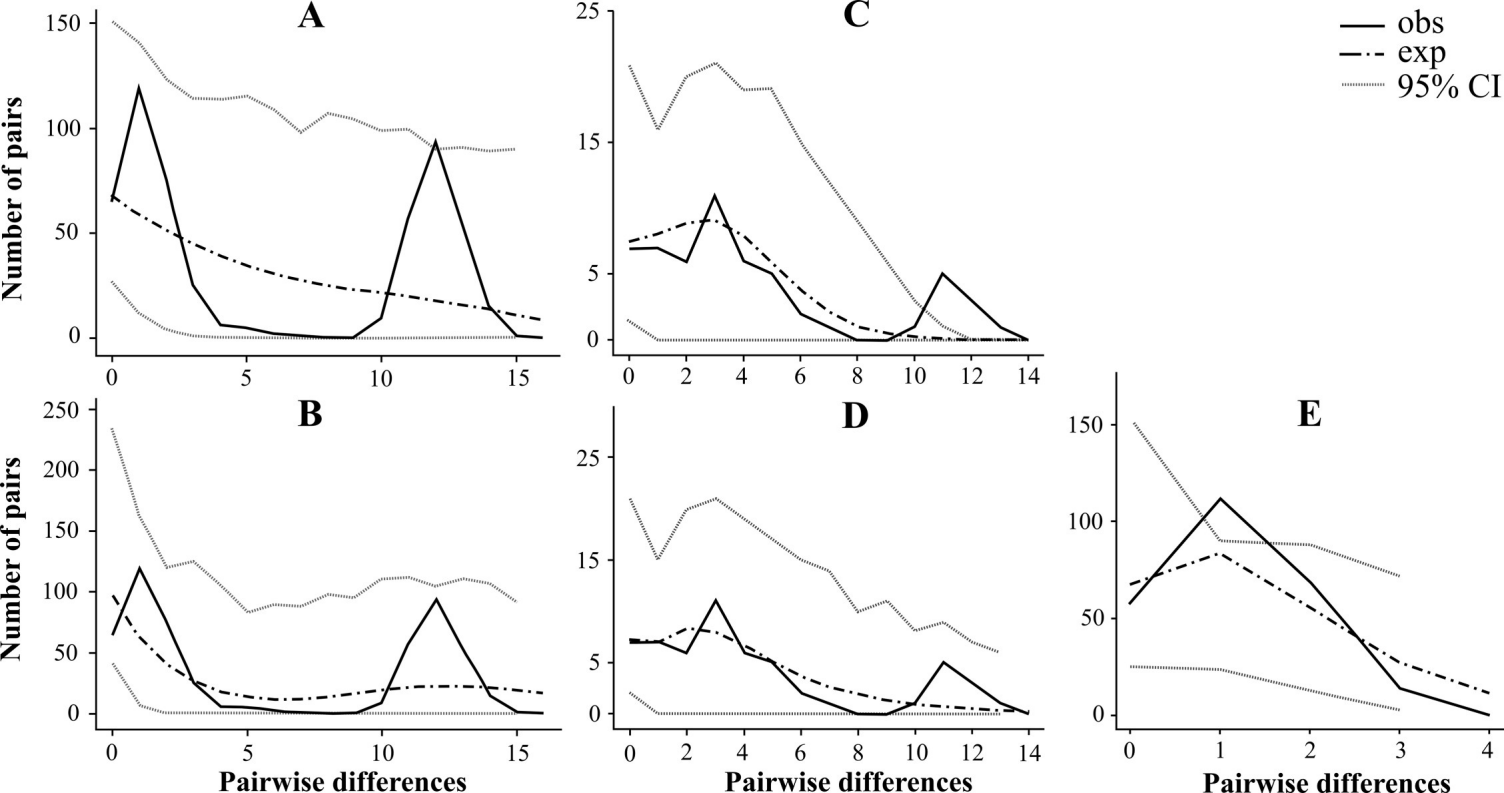
The mismatch distribution of the Siberian populations of *Daphnia longispina* based on the *12S* sequences. A and B, all Siberian populations; C and D, clade B; E, clade A. Black line represents the observed distribution. Dash and dot line and grey dotted lines represent expected distributions and the 95% credible interval for the sudden expansion model (A and C) and the spatial expansion model (B, D and E).

The mismatch distribution is bimodal for the *12S* sequences of all Siberian *D*. *longispina* populations (Fig 6A and 6B) what corresponds well to the spatial expansion model (SSD_obs_ = 0.05036). But MMD are different among two main clades: it is multimodal for the clade B (Fig 6C and 6D), but unimodal for the clade A (Fig 6E). Note that for clade A parameters of the models of demographic and spatial expanses are almost identical (Table 6). Therefore, two models could be used to explain the Siberian clade A expansion (SSD_obs_ = 0.01335), but the model of spatial expansion is apparently more acceptable for the Siberian clade B (SSD_obs_ = 0.01687).

## Discussion

### Notes on species distribution and co-occurrence

We confirm that *D*. *galeata* is widely distributed in water bodies of different types through all the range of North Palearctic, from Western Europe and European Russia through Western, Eastern Siberia and Transbaikalia to the Far East of Russia. In a few water bodies it co-occurs with *D*. *cristata*, *D*. *cucullata* (not described here) and *D*. *longispina*. We did not detect any cases of its co-occurrence with *D*. *dentifera*, although these two taxa co-exist in some water bodies in Japan and North America [38, 85]. In China *D*. *galeata*, *D*. *longispina* and *D*. *dentifera* also never co-occur [44, 50]. But it must be taken into consideration that *D*. *dentifera* was found here only in two mountain water bodies of Eastern Siberia, and a detailed study of this region could give a new information on the *D*. *dentifera* distribution and the cases of co-occurrence of this species with other taxa.

In contrast to *D*. *galeata*, we did not find any populations of *D*. *longispina* in Transbaikalia and the Far East of Russia; presumably the eastern border of this species distribution is the Yenisei River basin (Fig 1B), while all populations located east of this zone belong to *D*. *dentifera*. Three haplotypes of the former from the Baikal Lake basin and Mongolia are related to those from Japan and even North America. Such similarity with North American haplotypes was previously detected for those from Japan and China [38, 44]. We need to underline that all our findings of *D*. *dentifera* are from mountain lakes of the Baikal Lake basin. We can expect that further investigations will reveal other populations of *D*. *dentifera* in such mountain regions.

Previously Ishida & Taylor [48] revealed three clades of "*D*. *rosea*" (exactly corresponding to *D*. *longispina* s. lat.) across the Northern Eurasian range: European, Siberian and Japanese-American. If two first clades correspond to *D*. *longispina* s. str., the last one is *D*. *dentifera*. Ishida & Taylor [48] found that Siberian *D*. *longispina* are widely distributed in Siberia, east up to the Baikal Lake basin based on the *ND2* gene determination. Therefore, the whole Yenisei River basin, including Baikal Lake connected to the Yenisei River through the Angara River, is a transitory zone between *D*. *dentifera* (penetrating west up to 96°E) and *D*. *longispina* s. str. (penetrating east up to 104°E). According to our finding we believe that *D*. *longispina* and *D*. *dentifera* are vicarious species, although we did not study nuclear genes here, and can not detect possible hybrids in this zone, nor do we discuss the introgression pattern between *D*. *dentifera* and *D*. *galeata* [38, 48]. Such a transitory zone between western and eastern Palearctic phylogroups and taxa is previously known for all of the cladocerans studied for trans-Eurasian phylogeography [17, 18, 57].

### *12S* + *ND2* phylogeny

Phylogeny of the *D*. *longispina* complex, reconstructed here based on the sequences of the *12S* and *ND2* genes, in general agrees with previously published phylogenies [11, 12] and recent ideas on the group taxonomy [60]. Although a general topology of the trees based on *12S + ND2* and *12S* + *16S* genes (see [52, 53, 86] for the latter) is basically similar, we need to take into consideration a few differences between them.

In this study, *D*. *galeata*, *D*. *longispina* and *D*. *dentifera* form a moderately supported clade, which confirms their close relationships and monophyletic origin. But there are several divergent clades within the cluster corresponding to each taxon studied here. Some phylogroups of *D*. *galeata* and *D*. *longispina* are recorded in the remote regions of Siberia and the Far East only, which is, most probably, a sign of their long-term isolation. The presence of a high number of divergent haplotypes could be also a sign of recent speciation processes and cryptic diversity within this group. The latter is detected for the cladocerans of different families and genera using genetic methods [16–18] and requires further morphological studies with aim to find the morphological differences between the taxa within such groups.

We did not reveal distinct internal clusters within *D*. *longispina*, in contrast to previous *ND2* and *16S* phylogenetic reconstruction [48, 53] (our dataset on *D*. *dentifera* is too small for such an analysis). The *12S* and *ND2* haplotypes of *D*. *hyalina* do not form a distinct cluster, and could be regarded as a confirmation of the status of the latter as an ecological lake morph of *D*. *longispina* (following Petrusek *et al*. [11]). But the haplotypes from Hallstättersee Lake form a separate cluster adjoined to the Siberian clade B of *D*. *longispina* (albeit with a lack of statistical support). The latter could be regarded as an evidence for a species status of *D*. *hyalina* [30, 58, 60].

Finally, *12S* + *ND2* phylogeny confirms a basal position of undescribed taxon *D*. cf. *longispina* from the Chany Lake basin (the Ob River basin, Western Siberia) and its strong difference from *D*. *longispina* s. str., similarly to the data from other genes [52, 53]. Note that undescribed mitochondrial lineages from the *D*. *longispina* complex are recently revealed in other regions of Northern Eurasia [16]. To date, both endemic mitochondrial phylogroups and even endemic taxa are revealed in Eastern Siberia based on molecular methods in different cladoceran groups [17, 57], similarly to previously found endemics of the Far East [15, 28, 38].

### Phylogeography and demographic history

Our data on the polymorphism of the mitochondrial genes in European and Siberian populations of *D*. *galeata*, *D*. *longispina* and *D*. *dentifera* in general agree with those previously obtained for populations from other regions of Europe, Asia and North America [11, 36, 38, 44, 48–50]. Higher values of *H*_d_ and lower values of π in *D*. *galeata* and Siberian *D*. *longispina* clade A, that assumes a rapid population growth from an ancestral population with a low effective size, should be sufficient to restore the haplotype diversity through mutations, but insufficient for accumulation of significant differences in sequences [41]. But the pooled Siberian *D*. *longispina* populations, clade B of *D*. *longispina* and *D*. *dentifera* have a higher haplotype and nucleotide diversity of both mitochondrial genes in comparison with *D*. *galeata*. It could be a sign of a high stability and high effective size of their populations, or mixing of historically heterogeneous populations and distinct mitochondrial lineages presumably having different origin and evolutionary history [41]. We suppose that the second scenario is more realistic, because so many mitochondrial lineages are found in *D*. *longispina* and *D*. *dentifera* based on the *12S* [11, 16, 36, 44, 51, 52] and *16S* [53] sequences. Nevertheless, recently it has been shown, that such divergent mitochondrial lineages of the *D*. *longispina* complex can lack divergence of nuclear markers [40].

One of important global result of this study is related to the distribution of widespread and common *12S* haplotype of *D*. *galeata* in North Eurasia (i.e. in all studied Siberian regions). Such results based on more regional datasets [44, 87, 88] were vulnerable for criticism. This pattern of the haplotype distribution supports a hypothesis on a recent and fast expansion for most Siberian and Transbaikalian *D*. *galeata* populations. Together with the central *12S* haplotype (H5), the regions of Siberia, Russian Far East, Japan, China and Western Europe shared several peripheral haplotypes. But no haplotypes were shared between Siberia, Japan and North America; in addition, only a single haplotype from Siberia is closely adjacent to the haplotypes from these regions (H36, Fig 3I). Our data confirm conclusions on a wide distribution of some *12S* haplotypes of *D*. *galeata* in the Palearctic made by previous authors [38, 44, 86]. The same remarkable patterns in the *16S* haplotype distribution were also revealed previously [53]. Keeping into consideration (1) a very peculiar star-shaped structure of the *12S* haplotype network, (2) a unimodal mismatch distribution in Siberian populations based on the *12S* gene and (3) results of the Tajima’s *D* and Fu’s *F*_S_ neutrality tests we can confirm a recent expansion of *D*. *galeata* population through all the North Palearctic range. Note that our data on the *12S* polymorphism mainly corresponds to the "clade B" separated by Ishida & Taylor [49] based on the nuclear HSP gene (= "Assorted *D*. *galeata*" in their *ND2* tree), while their clade A ("New World *D*. *galeata*") is represented by a single branch (haplotypes H14 and H15) due to a rarity of *12S* sequences from North America.

In contrast, the *ND2* network has a more complicated structure with several less expressed star-shaped structures: the haplotypes form a network with five clades with their central haplotypes connected through hypothesized haplotypes m3-m5. The structure of *ND2* network and the multi-modal mismatch distribution for the Siberian *D*. *galeata* populations testify to a demographic equilibrium of them [78, 80, 89], at least in Siberia. Such discordance between two mitochondrial genes could be explained by different level of polymorphism of these two mitochondrial genes [41, 90, 91]. As it was earlier shown, mitochondrial genes in *Daphnia* evolve with different rates, and base-substitution mutation rates in *Daphnia* are higher then in most arthropods [12, 45, 92]. The different transition/transversion ratio, the rate of synonymous/nonsynonymous substitution, the number of polymorphic sites and total mtDNA sequence length, sample size, effective population size, etc. could be regarded as a reason of such discordance [93–95]. The *12S* gene is a more conserved region than the protein-encoding *ND2* gene. This is evidenced by a lower level of nucleotide and haplotype diversity (Table 1). Therefore, the patterns of deeper divergence between the studied populations of *D*. *galeata* would be more prevalent for the *ND2* gene.

The structure of the *12S* haplotype networks of *D*. *longispina*, the neutrality tests and mismatch distribution demonstrate a phylogeographic scenario in Siberia quite different from that in *D*. *galeata*. Positive (although not well-supported statistically) values of the Tajima’s *D* for both *12S* and *ND2* genes and Fu’s *F*_S_ for *ND2* gene for all Siberian populations and clades of *D*. *longispina* suggest a recent bottleneck effect and/or influence of the overdominance selection. Negative values of these tests (mainly for the *12S* gene) allow to reveal a tendency to numerous mutations with a low probability, what could be a sign of a recent population expansion and/or influence of negative (purifying) selecting. The unimodal mismatch distribution for clade A and multi-modal distribution for clade B testify their different evolution history. Hence, the star-like shape of the *12S* haplotype network together with values of the neutrality test and unimodal MMD more likely suggest a recent demographic expansion for clade A of *D*. *longispina*. In contrast, clade B exhibits different demographic history and support the demographic equilibrium of its populations. In general, the demographic history of the *D*. *dentifera* populations in Siberia is similar to that in *D*. *longispina*.

Using the equation τ = 2*ut* and based on the mean divergence rate of 2% per million years and assuming 3 generations per years, we can very roughly estimate the expansion time for the Siberian populations of *D*. *galeata* and *D*. *longispina*. The expansion time for *D*. *galeata* (with 95% confidence intervals) is about 101 325 (50 189–315 341) years ago based on the *12S* fragment and 493 524 (263 231–697 075) years ago based on the *ND2* fragment. The estimated expansion time for clade A is 125 947 (31 250–238 636) and for clade B is 169 508 (47 348–952 652).

There are numerous corroborations for pronounced errors of the estimations of divergence time for different mitochondrial lineages and species, especially keeping in mind the fact that there is no possibility to calibrate the molecular clocks for particular studies [18, 96, 97]. Here we tried to speculate on the demographic history of the Siberian *D*. *galeata* and *D*. *longispina* populations taking into account the geological events in this region during the Pleistocene.

During the coldest phases of Pleistocene, the northern flows of the large Siberian rivers was interrupted by a single continental glacier resulting in a considerable increase in the water level in the middle reaches, and appearance of huge proglacial (ice-dammed) lakes in the north [18, 54, 56, 98]. Distribution of the mountain glaciers in more southern regions of Central and East Siberia was correlated with the directions of the main airstreams in winter: the most extensive ice sheet was existed in the Altai-Sayan mountain area, it was gradually decreased in thickness from west to east. At that time such large lakes as Teletskoye (as well Tchuyskoye and Uymonskoye) were served as the refugia of freshwater fauna in Altai Mountains [56, 99]. Main refugia in the Yenisei River basin were situated in the Todzha and Darkhat Depressions having lakes with size significantly changing during Pleistocene glacial cycles [100]. These lakes have appeared repeatedly during the periods of a deglaciation and a high water level. Most recently such a lake in the Todzha Depression was formed about 10000–12000 years ago.

Southern Siberian lakes could be colonized by the daphniids already after the last deglaciation, i.e. around 10–15 000 yes ago, but such scenario seems to be less possible, i.e. keeping in mind the molecular clock estimations above. We think that the daphniids had a chance to survive in some refugia and such evolutionary lineages subsequently had an advantage as compared with other lineages as it is suggested by the "monopolization hypothesis" [101].

Detection of shared haplotypes in all Siberian clades is related with the high ability of the cladocerans to produce resting stages capable of a long-distance passive dispersion [102–104]. It is likely that the haplotypes of *D*. *longispina*, which at present compose the core of clade A, are the remains of the ancient fauna which were survived in some refugia during the glacial cycles. Taking into account the great age of the Cladocera [105], we could assume that several species of the *D*. *longispina* complex were widespread across whole the territory of Eurasia in pre-Pleistocene epochs. During the Pleistocene glacial cycles, their distribution ranges were repeatedly pulsed in size, sometimes they were restricted to small refugia. Similar scenarios could be also traced in the local whitefish populations in the water bodies of the Ob and Yenisei river basins [106]. Similar patterns of a postglacial expansion dating to the late Pleistocene were revealed for *Polyphemus* [15] and North American and Japanese *D*. *galeata* populations [48, 49]. The same was shown for the *D*. *longispina* populations in high altitudes Pyrenees and Tatras, which were also characterized by a restricted gene flow between populations and a strong founder effect [45, 107].

## Conclusions

Our results suggest that the evolution of closely related species in the same wide range (i.e. across whole North Eurasia) could follow different scenarios. In general, the described network structures, haplotype distribution patterns in different species, together with different *F*_ST_ values assumes significant differences in the evolutionary history of different species: the phylogeographic scenario was relatively similar in *D*. *dentifera* and *D*. *longispina*, but it was quite specific in *D*. *galeata*. Our genetic analysis of *D*. *galeata* population confirms its recent and fast expansion. In contrast, high haplotype diversity in the *D*. *dentifera* and *D*. *longispina* populations could be explained by the survival of different phylogroups in several glacial refugia located in Siberia. For all studied species, we detected in Siberian populations some unique haplotypes, maximum haplotype diversity is recorded in remote regions of Siberia–lakes of the Yenisei River, the Baikal basin and Transbaikalia, which requires further intensive studies of *Daphnia* genetic diversity. We believe that these Siberian regions could be a source of undescribed taxa of daphniids as well as a region of high diversity for other freshwater invertebrates.

## Supporting information

S1 Table Complete list of water bodies in which *Daphnia* species was studied (name, geographical position and abbreviation corresponding to Fig 2), number of sequenced individuals for each species and the GenBank accession numbers of *12S* and *ND2* sequences used in this study. (XLS)
